# Enhancing Safety Culture for Formal Caregivers in Long-Term Care: A Rapid Review

**DOI:** 10.1155/jonm/3887187

**Published:** 2025-07-25

**Authors:** Mathias Haeger, Sandra Garay, Kristin Krieger, Simon Eggert

**Affiliations:** Center for Quality in Care (ZQP), Berlin, Germany

**Keywords:** formal caregivers, long-term care, nursing homes, safety climate, safety culture

## Abstract

**Aim:** This study explores the latest intervention strategies, contributing factors, and measurement instruments aimed at enhancing safety culture in long-term care settings.

**Background:** A positive safety culture is associated with increased patient safety. While strategies to enhance safety culture are well documented in medical and clinical settings, evidence from the long-term care sector remains limited. Furthermore, the literature is heterogeneous regarding contributing factors and measurement approaches. Strengthening the understanding of how to enhance safety culture in long-term care could raise safety awareness among formal caregivers working with this highly vulnerable population of older adults.

**Methods:** A rapid integrative review was conducted to update our previous work on interventions. Relevant empirical and theoretical studies were retrieved from eight databases. The title, abstract, and full-text screening, as well as data extraction, were performed by two independent reviewers. Discrepancies were resolved through discussion with a third reviewer. Study quality was assessed using critical appraisal tools, and PRISMA guidelines were followed. Results were synthesized narratively.

**Results and Conclusion:** 17 studies were identified, including only one intervention study. Findings on interventions remain still heterogeneous, but several strategies and factors contributing to safety culture were identified. Some studies emphasized the benefits of critical incident reporting systems. Moreover, several instruments to assess safety culture are available without a known gold standard. Due to the short search period and potential biases within the included studies, conclusions should be drawn cautiously. Nevertheless, the review provides valuable insights into strategies, contributing factors, and measurement instruments that can inform the development of future intervention planning to improve safety culture in long-term care. Further research should aim to determine the most impactful contributing factors and design tailored interventions accordingly.

**Implications for Nursing Management:** Enhancing safety culture in long-term care settings requires a multifaceted approach. Key elements include staff training, implementation of reporting systems, and process optimization, supported by strong leadership engagement. Regular evaluations based on a suitable measurement instrument and a protracted timeframe are advisable to achieve a meaningful and sustainable cultural change.

## 1. Introduction

Healthcare is facing a global challenge in ensuring patient safety [1]. This concern, however, has not received the same level of attention in long-term care. The increasing life expectancy and demographic changes are adding to the burden on the care system. The complexity of care for older adults, including psychological and physiological disorders and extensive medications, requires appropriate qualifications to avoid adverse events [2]. A study revealed that nurse leaders identified a lack of knowledge in providing care as an important reason for safety concerns [3]. Safety and high-quality care are crucial for care-dependent people, formal caregivers, and relatives. Formal caregivers in in-home care or nursing homes can influence safety and the quality of care through a positive organizational culture [4, 5]. The construct of organizational culture includes safety culture [5], which is often defined as shared beliefs, values, competencies, and behaviors within a team targeting a reduction of safety-related problems [6–8]. In addition, a difference also lies between the terms “culture” and “climate,” as Churruca and colleagues (2023) explain: “Culture” is the deeper underlying construct of “climate” as its surface-level manifestation [4]. In previous publications, our group has referred to this perspective and defined safety climate as a kind of measurable snapshot of the underlying safety culture [7, 9]. However, it is impossible to separate both clearly. Thus, we focus on safety culture and imply the safety climate measurement.

Improving safety culture in long-term care settings holds the potential for significant positive outcomes. Previous studies have shown that an enhanced safety culture is associated with fewer adverse events [10, 11]. Moreover, higher scores in safety culture predict better quality performance indicators (i.e., reduced deficiency citations for healthcare and fewer substantiated complaints) in nursing homes [12]. An initial safety culture assessment in an organization plays an essential role in its further development, as it informs about the status quo of safety culture and provides the opportunity to shift the staff's attention towards it [13, 14]. There are numerous validated instruments to evaluate the safety culture in long-term care facilities. However, they are not readily applicable across countries. Known instruments are, for example, the Canadian Patient Safety Culture Survey (Can-PSCS) Tool [15], the Manchester Patient Safety Framework or “Culture is Key” [16], the Nursing Home Survey of Patient Safety Culture (NHSOPSC) [17], and the Survey on Resident Safety in Nursing Homes (NRS) [18]. A collection of instruments to assess safety culture in long-term care settings could be useful for researchers and nurse managers. Choosing the most appropriate instrument from a collection of possible instruments (e.g., regarding setting, objective, and length) could serve as a starting point to evaluate the facilities' safety culture. Based on the assessment result, this creates the opportunity to decide which areas of safety culture to target. It should also be noted that simply conducting a safety culture survey could already impact it [14, 19].

Literature indicates that, for example, interventions to reduce stress and professional burnout, the implementation of a registry or reporting system of incidents/adverse events, staff training, or the collegial exchange of experiences might be beneficial for improving the safety culture in long-term care facilities [7, 20]. A recent review highlighted various published approaches (*n* = 57 strategies) to support management and staff in strengthening patient safety culture [21]. The authors divided these strategies into two categories: recommendations (i.e., focusing on teamwork, communication, and leadership) and actions (i.e., active participation and commitment to safety practices). This demonstrates that many factors could be considered in the context of safety culture interventions, which play into the fact that safety culture is a complex construct.

Much new research emerged on the topic, since the conclusions of our latest work were based on the literature search from 2020. At the same time, long-term care providers depend on the latest evidence to implement and sustain a positive safety culture in their organizations. Because of that, we undertook this update of previous work on safety culture interventions for nursing professionals in long-term care and an extension of our work regarding the measurement and contributing factors of safety culture. We therefore aimed to collect up-to-date evidence on methods linked to safety culture in the long-term care setting. The research was guided by the primary research question:

1. Which studies published since May 2020 describe interventions aimed to enhance safety culture in long-term care on a team or facility level?

The secondary research questions addressed factors associated with safety culture in long-term care:

2. Have there been new instruments for assessing the safety climate on the team or facility level in long-term care since May 2020?
3. What implications exist for reporting systems from intervention studies on safety culture in long-term care published since May 2020?
4. Which factors have a measurable or subjective reported impact on safety culture in long-term care at the team or facility level, according to the intervention studies published since May 2020?

## 2. Methods

### 2.1. Design

This rapid review adopted an integrative approach to examine the context, including diverse study designs [22–24]. The five-stage integrative review method was followed: problem identification, literature search, data evaluation, data analysis, and presentation [25]. The Preferred Reporting Items for Systematic Reviews and Meta-Analyses (PRISMA) guidelines were followed for the search strategy and the reporting of results, including precise research questions, a systematic search, screening and selection based on defined inclusion/exclusion criteria, data extraction, and synthesis as well as analysis of evidence [26]. Since this is an update of a previous study, we included studies published between 2020 and 2024. This review was preregistered in PROSPERO with the following ID: CRD42024570096.

### 2.2. Search Strategy

We included studies from PubMed (MEDLINE database) and EBSCO (including databases APA PsycArticles, APA PsycINFO, CINAHL, ERIC, PSYNDEX Literature with PSYNDEX Tests, and SocINDEX). Additional literature was identified by forward citation tracking of eligible studies and intervention studies from our previous work [7]. We focused on three areas of interest combined with an AND-operator: (I) the population (ambulatory care OR outpatient care OR agencies, home care OR long-term care OR nursing homes) as MeSH-term or title/abstract search, (II) interventions/concepts (training^∗^ OR education OR program^∗^ OR method^∗^), and (III) the context ((patient AND safe^∗^) OR (safe^∗^ AND culture) OR (risk AND manage^∗^) OR (safe^∗^ AND manage^∗^) OR (error AND manage^∗^) OR (safe^∗^ AND climate) OR (safe^∗^ AND measure^∗^) OR (medi^∗^ AND error) OR (nurs^∗^ AND error) OR (adverse AND event)) as title/abstract search. A more detailed example of our search strategy, including the syntax, is available on the PROSPERO registry. For all studies, the following inclusion and exclusion criteria were applied (Table 1).

### 2.3. Review Process

All studies were transferred from the databases to the tool Covidence for the title, abstract, and full-text screening. The first 50 studies in the title and abstract screening were used as exercises and discussed within the research team after an independent review (SG, KK, and MH). In the process, each study was screened and later extracted independently by two researchers (SG and MH). A third reviewer (KK) checked each conflict.

### 2.4. Data Extraction

The tool Covidence was also used for data extraction, including exporting to Microsoft Excel. Extracted data included the study objective and design, country, methods, population/setting, measurements/tools, a description of concepts/interventions, and results.

### 2.5. Assessment of Methodology/Risk of Bias (RoB)

Two reviewers (KK and MH) independently judged the RoB in relevant results, and disagreements were discussed until a consensus was reached. Characteristics of interest were, for example, randomization, confounding, or missing outcome data. Since we included different study designs, we used the appropriate assessment tools: RoB-2 tool for randomized controlled trials [27], ROBIS for reviews [28], AXIS for cross-sectional studies [29], and the JBI checklist for qualitative studies [30].

### 2.6. Data Analysis and Synthesis

The extracted data and the RoB assessment were reported descriptively with a narrative presentation of the overall results structured by the research questions.

## 3. Results

### 3.1. Descriptive Results

**Figure 1:**
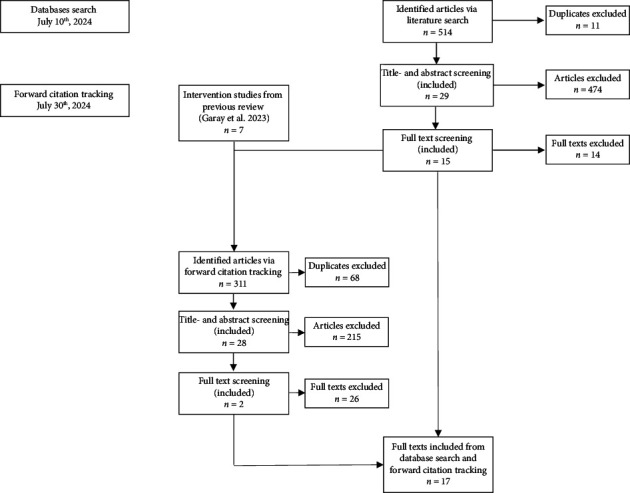
Flowchart of the study selection process.

A total of 825 studies were identified via database search and citation tracking. We excluded 79 duplicates, 689 studies in the title and abstract screening, and 40 more in the full-text screening. Finally, 17 studies were eligible regarding our research questions (Figure 1). All studies included were conducted in nursing homes or residential care facilities. No study from in-home care agencies or semiresidential facilities was included. Some studies showed overlaps regarding our research questions; thus, we found one intervention study, seven studies that mentioned new or updated instruments, four studies that discussed implications on Critical Incident Reporting Systems (CIRS), and ten studies that described contributing factors to safety culture.  Table 2 presents the characteristics of the included studies.

### 3.2. RoB Assessment

Most studies in this review were cross-sectional trials (*n* = 10). The RoB estimations in the AXIS tool ranged from 11 to 19 positive out of 20 possible answers, with a mean value of 16. In detail, more than half of the included studies did not justify their sample size [35, 37, 38, 41, 43, 45], did not address or categorize nonresponders [32, 34, 35, 37, 38, 41, 43, 46], and offered no information on nonresponders [32, 35, 37, 38, 41, 43, 46]. In six studies, the selection process was not likely to select participants that were representative of the target population [34, 35, 38, 41, 43, 46]. The qualitative studies (*n* = 3), assessed using the JBI, showed a mean of 8 out of 10 possible positive answers. Two studies did not address the role of the researcher in the process (own cultural and theoretical orientation) and the representation of participants and their voices [31, 47]. The reviews (*n* = 3) were assessed with ROBIS. Two showed a low RoB [33, 40], and one review did not adequately address the potential RoB from the study identification and selection process and thus was rated with an overall high RoB [39]. The RCT was rated at a high RoB, since one domain (measurement of the outcome) showed a high RoB [44].

### 3.3. Implications to Enhance Safety Culture

#### 3.3.1. Interventions to Enhance Safety Culture

We identified one intervention study that implemented a training package on safety culture in French nursing homes [44]. The package included a strategy to report and analyze adverse events associated with care, including clear definitions, examples, reporting tools, and a steering group responsible for analyzing and defining an action plan. Nursing homes were provided with methodological support given in four different sessions to raise awareness of taking ownership of the project, raise awareness among nursing home staff to report events, train the steering group on how to structure their work, analyze adverse events, and observe how the steering group handled critical adverse events. The intervention showed only for the seventh dimension (“Feedback and communication about incidents”) of the NHSOPSC a significant improvement from pre to post [44].

#### 3.3.2. Instruments to Assess Safety Culture

Seven studies were identified which reported or used instruments to measure safety culture in nursing homes: The CLC Employee Survey of Attitudes about Resident Safety (CESARS) [36–38], the Korean Patient Safety Culture Scale for long-term care facilities [34], the Adverse Event Reporting Obstacle Scale (AEROS) [32], the Occupational Health and Safety Council Safety Climate Questionnaire [39], and the Modified Stanford Patient Safety Culture Survey Instrument (MSI) [33]. All instruments reported acceptable validity and reliability. These instruments are new, since others (i.e., NHSOPSC) were already known from our previous review [7] and a report on safety culture [9]. In these previous studies, we identified the NHSOPSC, Culture is Key, Can-PSCS, Safety Attitudes Questionnaire (SAQ), and the NRS. All these instruments examine safety culture in residential or in-home care and therefore show some overlaps; however, we cannot indicate which instrument is most suitable.

#### 3.3.3. Implications for the Implementation of Critical Incident Reporting Systems

We included four studies that used or described a critical incident reporting system and mentioned facilitators or barriers. It should be noted that there is currently no standard system to report adverse events or incidents in long-term care settings [40]. Scott et al. gave an overview of existing systems, captured types of incidents commonly reported, and highlighted that more standardized systems might improve cross-organizational learning. The authors also argued that digital reporting systems should be favored, as they reduce the workload of paper systems (i.e., data entry and evaluation) [40]. Another study described a facilitating effect of patient safety culture on reporting attitudes to adverse events [46]. Moreover, their results indicate that individual characteristics such as reporting awareness, education level, and working experience significantly influenced the staff's reporting attitude. Wang et al. concluded that nurse managers should simplify the reporting procedures to enable staff with a lower educational level to report incidents. Finally, the authors from one study noted that a lesson learned from incident management was to emphasize processing reported incidents at the organizational level transparently and to initiate solutions openly with the team to avoid the reoccurrence of safety incidents [42]. The authors assumed that not addressing lessons learned for the organization could lead to isolating learnings to only the individual employee rather than an opportunity for the whole organization to improve safety practices.

Three studies describe barriers to the implementation of a reporting system [35, 42, 46]. One study mentioned a high workload as a possible barrier to adverse event reporting. Especially when nurses lack time to complete their tasks in direct care, an additional duty, such as filling out a reporting for, may be neglected [46]. The authors also described hierarchical reporting and working conditions as a barrier since, for example, nurse assistants typically relay information orally to the head nurse and do not use a reporting system on their own [46]. Another study referred to nurses' negative emotions such as shame and guilt, a loss of confidence after experiencing conflicts or repercussions, and a lack of support from peers after reporting incidents [42]. Moreover, an examination of professionals' views on safety culture and incident reporting systems showed that the staff felt that the organization was neither learning from the system nor providing enough feedback [35]. The authors also assumed that there might be a reporting difference depending on the kind of incidents, i.e., falls that happened in the absence of a nurse might be easier to report since professionals are not usually held responsible for them.

#### 3.3.4. Contributors to Safety Culture

In sum, 10 studies reported factors that contributed to safety culture. The identified factors can be grouped into organizational, care process–related, or staff-related aspects. At the organizational level, the included studies identified the implementation of training in safety culture practices (“risk management approach”) as well as an already initiated quality approach and a steering group that demonstrated leadership [44], organizational readiness to change at different levels (i.e., coworkers, supervisors, and senior managers) [36], or the facility scale (i.e., larger facilities), ownership (i.e. state-owned), and whether it was an integrated care institution which provided medical and aged care services as opposed to only aged care services [32] as aspects with a positive influence on safety culture. However, there were heterogeneous results regarding facility scale since another study showed that larger facilities and being attached to a hospital resulted in perceptions of a lower safety culture [43]. Regarding contributors to safety culture on the care process level, one study highlighted the division of roles according to expertise, smooth information sharing, open communication, as well as an active participation in the development of care-related rules and respectful relationships between professions as beneficial for safety culture [47]. Another study pointed out that good reporting management of adverse events might improve safety culture [32]. Besides that, the authors described the reported frequency of concerns regarding patient safety, the occurrence of actual adverse events in the departments, and a punitive atmosphere as further influencing aspects [32]. Finally, there are staff-related aspects that might improve safety culture. With this, two studies showed that the length of service (work experience) [37] as well as staff turnover of registered nurses and certified nurse assistants [45], had a negative effect on the perception of safety culture. To navigate that, Quach et al. (2021) suggested pairing experienced workers with newer ones to narrow potential knowledge gaps and increase collaboration. In addition, huddles, team meetings, and organizational initiatives could facilitate recognizing and leveraging experienced workers' accumulated safety knowledge [37]. Higher reporting attitudes and reporting awareness of adverse events were associated with a higher perception of safety culture [46]. Furthermore, two studies linked a positive safety culture to leadership behavior [31, 41]. In detail, Seljemo et al. reported that transformational leadership, defined by idealized influence, inspirational motivation, intellectual stimulation, and individualized consideration, was linked to a better safety culture. Engle et al. described leadership support, communication, and responsiveness as core influential factors in the staff's perceived safety culture. The authors stated that in facilities with a higher safety culture, staff had the sense that people in positions above them in the hierarchy were open to conversations, were approachable, and were physically present to have face-to-face conversations; moreover, they found their nurse managers and senior managers to be supportive and approachable [31]. On an individual staff-related level, skill utilization and job demands (i.e., work pace and emotional workload) influenced safety culture [41].

## 4. Discussion

This review updates previous work, focusing on interventions to improve safety culture in long-term care. Between 2020 and 2024, we identified 17 studies, including only one intervention study. Other aspects of interest were instruments for measuring safety culture, implications for implementing CIRS, and factors that might influence safety culture in long-term care settings. Even if the findings on new intervention studies are marginal, this integrative review provides a beneficial overview for the development of future intervention studies and research projects.

The intervention study implemented a training package to improve the reporting and analysis of care-related adverse events and safety culture in nursing homes. It was shown that the 7^th^ dimension of the NHSOPSC, namely, “feedback and communication about incidents,” improved in the facilities [44]. It seems appropriate, as some training content (i.e., raising awareness, available tools, and empowering some professionals to analyze events and provide feedback) was also related to this area. Such intervention content is partly in line with previous studies that, for example, intended to facilitate the collegial exchange of experiences and learnings and create an environment of reporting and learning from mistakes [7]. Overall, however, the approaches and results of intervention studies are too heterogeneous to allow targeted comparisons and to identify the most effective interventions. Thus, as previous studies stated [20, 48], further intervention research is still required.

To give further recommendations on intervention designs, we also examined existing measurement instruments for safety culture, CIRS (due to their proximity to safety culture), and factors influencing safety culture. Some of the instruments, several of which have been validated in different languages, were already known from our previous work. The current research adds the CESARS, the Korean Patient Safety Culture Scale for long-term care facilities, the AEROS, the Occupational Health and Safety Council Safety Climate Questionnaire, and the MSI. These instruments were identified in response to our second research question and serve to broaden the overview of available tools. However, it must be noted that this overview may not be exhaustive, as the search strategies of both reviews were not specifically tailored to identify measurement instruments. Therefore, the intention here is to offer guidance on potentially useful instruments for assessing safety culture in long-term care settings. Although all included instruments were described as validated, no single one has been established as a “gold standard” or received official recommendation, unlike in the hospital setting, where, for example, the European Commission has issued guidance [49]. One recommended instrument is the Hospital Survey of Patient Safety Culture (HSOPSC) [50], which has also been adapted for nursing homes and has recently been validated in a short version [51]. This short version focuses on four key dimensions of safety culture: (i) safety improvement actions, (ii) teamwork, (iii) information flow, and (iv) management support. Other instruments may emphasize similar or distinct areas, such as responses to mistakes and errors. In practice, the decision in favor or against a particular instrument therefore depends on which dimensions of safety culture should be addressed and evaluated in the context of the intervention. The results of an initial safety culture survey can help identify areas with room for improvement before an intervention is implemented. Follow-up assessments can then indicate whether the steps taken had an impact on the previously identified dimension. In addition to selecting a suitable instrument, incorporating patient safety quality indicators, such as falls, pressure ulcers, or use of physical restraints, may enhance evaluation efforts [33]. These indicators allow to reveal changes in the quality of care as a proxy for improvements in safety culture. Combined feedback from measurement tools and quality indicators can presumably improve the effectiveness of interventions in safety culture.

In addition to the instruments described above, CIRS can contribute to safety culture by recording and evaluating safety-related incidents. Four of the included studies examined correlations between the use of reporting systems and safety culture [35, 40, 42, 46]. It was shown that safety culture is related to the reporting attitude of adverse events [46], that organizational learning processes can be improved [42], and that the data from reported incidents allow conclusions to be drawn about safety culture [35]. The link between reporting systems and safety culture can be used to learn specifically from incidents by reviewing and discussing them with the team. A standardized system used in different facilities could also be valuable for cross-organizational learning [40]. Regarding data evaluation and feedback processes, a digital system would be preferable to an analog one (i.e., paper and pencil) [40]. In addition, it could be helpful to use reporting and learning systems or appropriate training as an intervention approach to improve safety culture, as was intended in the intervention study mentioned above [44]. However, reporting systems not only affect an organization's safety culture but their outcomes also depend on the preexisting safety culture on site: WHO describes that incident reporting and learning systems can only be meaningfully advanced based on a cultural change toward a positive safety culture [1].

Besides reporting systems or measurement instruments, which in themselves can already positively influence safety culture [19], other factors can be intervention content. Therefore, it is essential to note that safety culture is a complex construct that is part of the organizational culture [5, 52]. Thus, it could be recommended that interventions address several factors rather than a single one. These factors can, for example, affect the organization, the care process, and the staff. We differentiated the included studies into these areas, whereby some overlaps also become clear: For example, the “organizational readiness to change” [36] can not only address single professionals but can also be understood as overarching for the entire organization. Furthermore, open communication, better information sharing, or good incident reporting management [32, 47] are process-related factors that could also be addressed through personal training of nursing managers [31, 41]. Therefore, adapting a process should be linked to staff training about the implementation. Such overlaps on individual and organizational levels show that factors influencing the safety culture often cannot be clearly separated, which suggests intervention approaches connecting the different levels. This is in line with a recent study that pointed out that a combination of different methods (i.e., communication, teamwork, and active leadership) might be beneficial for strengthening safety culture in the healthcare sector [21].

### 4.1. Limitations

Our study has some limitations that should be discussed. The included RCT was rated at a high RoB, and therefore, the significant effects regarding the 7^th^ dimension of the NHSOPSC should be considered carefully. This is comparable to our previous study, in which all included studies showed an overall critical RoB [7]. Therefore, the effect of the contents or strategies of all intervention studies included in our last reviews must be considered cautiously and should only be used as an indication for further studies. The overall RoB from the other included studies was, in sum, moderate, with a small RoB in five studies [33, 36, 40, 42, 45]. However, other limitations must be considered even if the risk is lower in the nonintervention studies. We have limited the search period for our secondary research questions to restrict the literature included. Nevertheless, this point limits the informative value: Studies on measurement instruments and reporting systems may be missing, or important factors influencing the safety culture may not be taken into account. Furthermore, we excluded short-term care, palliative care, psychiatric care, the care of younger people, and care of people with disabilities, which limits the significance of our results in the long-term care of older adults. Finally, we also excluded the clinical care setting, as, in our view, the results cannot thoroughly be transferred between the settings, especially as other studies indicate setting-related differences in the perception of safety culture [35].

### 4.2. Implications for Nursing Management

To effectively enhance safety culture in long-term care settings, nursing management should adopt a multifaceted strategy that integrates organizational development and staff support. A strong safety culture requires conscious and sustained leadership efforts to create an environment in which safety is understood as a shared responsibility, not a source of blame. Nurse leaders play a central role in fostering open communication and psychological safety, encouraging staff engagement in incident reporting and ensuring that lessons learned are reflected upon at the team level without fear of negative consequences. Regular training that promotes team-based reflection, simplified reporting procedures accessible across education levels, and emotional support after adverse events are key elements of such an approach. In addition, nurse managers should make use of validated safety culture measurement instruments to identify areas for improvement and monitor progress over time. Suggestions for appropriate instruments can be found in the results section and previous publications. Importantly, efforts to strengthen safety culture should be understood as long-term processes that ultimately support high-quality care.

## 5. Conclusion

Taking the current findings together with those of our previous review, several key conclusions can be drawn. First, intervention studies targeting safety culture in long-term care remain scarce, particularly in residential and home care settings. Since our last review, we identified only one additional intervention study in these areas. Second, the content and approaches of existing interventions remain highly heterogeneous. This diversity is understandable, given the multitude of factors that appear to influence safety culture, and the current lack of evidence regarding which of these factors are most critical. Nevertheless, each of the studies reviewed offers valuable insights into the nature, and collectively, they lay the groundwork for the development of targeted interventions. In this context, our reviews provide a foundation for informed intervention planning, including practical guidance on strategies, contributing factors, measurement instruments, and the role of reporting systems. For instance, the findings can help identify which dimensions of safety culture require improvement (based on measurement data) and guide the selection of appropriate intervention strategies to address relevant influencing factors. Since safety culture is a complex construct influenced by multiple overlapping factors, interventions are likely to be effective when they target several layers of safety culture simultaneously. In addition, the relationship between safety culture and reporting systems deserves further exploration, not least, because reporting systems themselves may serve as intervention tools. This potential has been highlighted in previous research [40] and warrants closer investigation. Finally, intervention planning must also carefully consider how to measure outcomes, given the absence of a gold standard for assessing safety culture. In our view, future research should prioritize two aims: (1) identifying the factors with the greatest influence on safety culture and (2) designing specific, evidence-based interventions targeting these factors. It is important to recognize, however, that developing a strong safety culture is a long-term process that requires sustained efforts and time.

## Figures and Tables

**Table 1:** Overview of the applied inclusion and exclusion criteria.

Inclusion	Exclusion
- Published between 2020–2024	- Studies that focus on informal caregivers, patients, or people in need of care
- English or German language	- Settings: pediatric, psychiatric, clinical, intensive, palliative, or assisted living, where no professional care is provided regularly
- Residential or outpatient long-term care setting	- Interventions that did not aim to enhance safety culture/climate, psychologically safe work environment, or processes of critical events
- Evaluations of formal caregivers (i.e., professional nurses)	- Studies related to COVID-19
- Interventions/trainings/educational sessions for formal caregivers on aspects of safety culture/climate or perceptions and values in relation to risks within the organization	- Unpublished studies or protocols
- Studies should address the primary or secondary research questions	
- Study design: evaluation studies, cohort studies, RCTs, controlled trials, case-control studies, clinical trials, multicenter studies, as well as reviews, meta-analyses, or guidelines	

**Table 2:** Descriptive data for all included studies.

Study	Year	Country	Design	Population	Aim/research question	Data collection	Most relevant results
Engle et al. [31]	2023	USA	Qualitative	Nursing home staff	To understand staff perceptions of resident safety	Interviews	Communication, leadership support, and responsiveness were identified as important aspects of a higher safety climate in nursing homes
He et al. [32]	2020	China	Cross-sectional	Nursing home staff	To explore factors associated with PSC and its relationship with obstacles to adverse event reporting	NHSOPSC, AEROS, demographic background	The multivariate regression model of patient safety culture showed a negative impact of privately owned facilities (Beta = −0.369, *p* < 0.001), reporting management (Beta = −0.330, *p* < 0.001), whether adverse events had occurred in departments (Beta = −0.139, *p*=0.001), punitive atmosphere (Beta = −0.101, *p*=0.044), and a positive association with increasing facility scale (Beta = 0.352, *p* < 0.001), whether it is an integrated care institution (Beta = 0.190, *p*=0.006), and frequency of concern about patient safety (Beta = 0.140, *p*=0.001); results showed a negative association between PSC and obstacles to adverse event reporting
Kim et al. [33]	2022	Republic of Korea	Review		To identify tools that measure patient safety and identify factors affecting patient safety		Most tools that measure patient safety were related to PSC and employees' attitudes; higher PSC scores were associated with lower medical defects; nursing homes scored lower than hospitals in PSC
Lee and Cho [34]	2022	Republic of Korea	Cross-sectional	Registered nurses and nursing aides	To identify the relationship between PSC and safety activities and to explore influencing factors	Korean patient safety culture scale for LTC facilities	A significant correlation between PSC and patient safety activities; factors influencing patient safety activities among RNs and NAs in LTC facilities were as follows: RNs (Î^2^ = 0.377, *p* < 0.001), organizational system of PSC (Î^2^ = 0.314, *p* < 0.010), and work shift type (fixed night shift, on-call, 24-h shift) (Î^2^ = −0.264, *p* = 0.004), which explained about 36.0% of total variance (*F* = 5.69, *p* < 0.001)
Liukka et al. [35]	2021	Finland	Cross-sectional	Manager, registered nurses, practical nurses	To examine whether PSC perceptions differ between managers and other professionals as well as between LTC and acute care	HSOPSC/NHSOPSC, demographic background, data from the incidence reporting system	Half of all reported incidents were accidents, commonly falls, presuming that falls are easier to report; managers estimate PSC more positively than other professionals; worst results were in composites that are related to managers' expectations and actions to promote and support PSC; therefore, staff felt that the organization is neither learning from the reported mistakes nor providing enough feedback
Quach et al. [36]	2021a	USA	Cross-sectional	CLC staff members	To examine the association of organizational readiness to change with safety culture	CESARS, ORCA, data about the structure and process of care	Organizational readiness to change was associated with safety climate; thus, it might be a requisite aspect for safety climate improvements; readiness to change in nursing homes (i.e., openness to change in frontline staff, opinion leaders, and senior managers and by communications between them) was an essential ingredient in a strong safety climate
Quach et al. [37]	2021b	USA	Cross-sectional	CLC staff members	To examine whether longer service is associated with more or with less positive safety climate perceptions	CESARS, staff, and facility characteristics	Staff with longer lengths of service perceived their safety climate less positively regarding their supervisor's commitment to safety, interactions with coworkers around safety, and the global ratings of their CLC than staff with shorter length of service; staff with longer service may have more knowledge of safety, making them especially critical of their supervisors, coworkers, and facility overall in terms of safety performance
Quach et al. [38]	2021c	USA	Cross-sectional	CLC staff members	To examine whether a more positive safety climate would be associated with lower rates of adverse events	Data of adverse events, CESARS, ORCA	Supervisor commitment to safety, environmental safety, and global rating of the CLC predicted four adverse events, which suggests that safety climate may be an organizational pathway to lower multiple adverse events; more positive ratings of supervisor commitment to safety were associated with lower rates of adverse events; associations between global ratings of the CLC and catheter use were in an unexpected direction (more positive ratings were associated with higher rates of catheter use)
Rand et al. [39]	2021	United Kingdom	Review		To identify measures that could be used as indicators of safety for quality monitoring and improvement		PSC was lower in nursing homes than in hospitals, with lower levels of learning from errors, less open communication, and a blaming or punitive culture among staff; work environment may influence resident safety outcomes more than the traits of individual care staff; workplace indicators may also be important sources of additional information to support the interpretation of harm-based indicators
Scott et al. [40]	2024	United Kingdom	Review		To identify the safety incident reporting systems and processes used within care homes to capture staff reports of safety incidents and to note the types as well as the characteristics of safety incidents captured by safety incident reporting systems		Nurses were responsible for incident reporting more than any other profession or role, but it was unclear why nurses were most involved in incident reporting; there is no standard reporting system, however, many share common features; captured data are still heterogeneous; most frequent reported incident categories were patient behavior, clinical process/procedure, documentation, medication/intravenous fluids, and falls, which broadly reflects previous evidence; the most often identified factor in reducing risk was to improve safety culture; patient, staff, and organizational factors also contribute to safety incidents; regarding patients, cognition was the highest contributing factor to incidence occurrence
Seljemo et al. [41]	2020	Norway	Cross-sectional	Nursing home professionals	To assess the association between transformational leadership, job resource, job demand scores, PSC, and employees' overall perception of patient safety in nursing homes	NHSOPSC, GTL, SIMPH	Transformational leadership was identified as a strong predictor of PSC and the overall perception of patient safety; the only additional significant predictor was emotional workload, predicting perception of patient safety; job demands and job resources showed no significant improvement in explained variance in any of the final models
Serre et al. [42]	2022	Canada	Qualitative	Registered nurses, registered practical nurses, and personal support workers	To describe nurses' experiences with patient safety incident management involving residents living in LTC	Observations and interviews	Three main categories emerged in relation to participants' experiences with managing PSIs: (1) commitment to resident safety, (2) workplace culture, and (3) emotional reaction; furthermore, it was noted that PSIs were seen as opportunities for improving safety practices such as engaging in reflection postincident and learning with others; workplace culture captures the unit and team-level factors that influence nurses' PSI management experiences in LTC; participants also noted helpful working conditions when resources, such as equipment (e.g., for taking vital signs) and protocols for managing particular PSIs, were available
Teigné et al. [43]	2021	France	Cross-sectional	Nursing home professionals	To evaluate the level of SC and to identify factors that could predict SC scores	NHSOPSC-F (French version), descriptive variables for NHs	Compliance with procedures (Dimension 5) was lower among NHs with over 80 beds, those with a qualified, in-house risk manager, and those that used an external quality and RM provider; staffing (Dimension 6) was lower among NHs that had initiated a quality improvement approach; feedback and communication about incidents (Dimension 7) were lower for NH attached to a hospital than for those that were part of a group; there is also a clear link between perceptions of the difficulty of complying with procedures when the workload was high, and staffing levels were low
Teigné et al. [44]	2022	France	RCT	Nursing home professionals	To study the impact of a training package on safety culture and identify drivers for improvements	NHSOPSC-F, descriptive variables for NHs	Significant improvement in the dimension of “feedback and communication about incidents” of the NHSOPSC-F, drivers were found in Dimension 1 (percentage of members of the steering group who showed leadership, and in NHs with an active quality improvement approach), Dimension 3 (NH had an established policy of ongoing improvement in quality and RM), Dimension 4 (when the NH was hospital based, but not when it was independent, or when fewer staff wanted to use the knowledge acquired), and Dimension 6 (as a function of the percentage of members of the steering group who wanted to use the knowledge they had acquired, when there was an active quality improvement approach, and when the dependency score increased)
Temkin-Greener et al. [45]	2020	USA	Cross-sectional	Nursing homes (directors of nursing, facility leaders, and unit nursing leaders)	To examine the association between turnover of RNs and CNAs and perceived PSC in NHs	NHSOPSC, questions on staff turnover, NH characteristics	Low turnover of RNs and CNAs showed a strong, statistically significant, and positive association with PSC, both for the overall score and individually for most of the 12 domains; the PSC domains of teamwork, staffing, and training/skills appeared to be mainly related to CNA turnover but not to RN; PSC domains focusing on collaboration across disciplines and roles, such as compliance with procedures, handoffs, communication openness, and organizational learning, appeared to be equally associated with CNA and RN turnover, suggesting that the effect of turnover on PSC domains depends on the position that the person leaving had
Wang et al. [46]	2024	China	Cross-sectional	Nursing home staff	To explore the current status of AEs' reporting attitude, and the individual and organizational factors among nursing staff in nursing homes	Sociographic data, facility data, incident reporting attitude scale, adverse event reporting awareness scale, NHSOPSC	Strongest predictors for AE reporting attitude were organizational characteristics, particularly safety culture and AE reporting awareness; other predictors were individual characteristics such as education level and work experience
Yamamoto et al. [47]	2023	Japan	Qualitative	Nurses and caregivers	To identify the elements of safety behavior that allow nurses and caregivers to develop collaboratively a culture of safety in nursing homes	Interviews	Identified aspects for building a safety culture were open communication–promoting work environment, smooth information sharing, and role sharing based on expertise; mutual respect, acceptance of opinions, and good information sharing were described as beneficial; the development of an infrastructure of information sharing and the demonstration of collaborative skills were essential for building a safety culture

Abbreviations: AE, adverse events; AEROS, Adverse Event Reporting Obstacle Scale; CESARS, CLC Employee Survey of Attitudes about Resident Safety; CLC, community living center; CNA, certified nurse assistants; GTL, Global Transformational Leadership Scale; LTC, long-term care; NA, nurse aides; NH, nursing home; NHSOPSC, Nursing Home Survey of Patient Safety Culture; ORCA, organizational readiness to change assessment; PSC, patient safety culture; PSI, patient safety incidents; RM, risk management; RNs, registered nurses; SIMPH, short inventory to monitor psychosocial hazards.

## Data Availability

No datasets were generated or analyzed during the current study.

## References

[1] World Health Organization-WHO (2021). *Global Patient Safety Action Plan 2021–2030: Towards Eliminating Avoidable Harm in Health Care*.

[2] Ewers M., Lehmann Y., Jacobs K., Kuhlmey A., Greß S., Klauber J., Schwinger A. (2020). Hochschulisch Qualifizierte Pflegende in Der Langzeitversorgung?! University-Qualified Nurses in Long-Term Care?!. *Pflege-Report 2019: Mehr Personal in Der Langzeitpflege - Aber Woher?*.

[3] Eggert S., Sulmann D., Teubner C. (2020). Sicherheitskultur in Der Ambulanten Pflege. *ZQP-analyse*.

[4] Churruca K., Falkland E., Saba M., Ellis L. A., Braithwaite J. (2023). An Integrative Review of Research Evaluating Organisational Culture in Residential Aged Care Facilities. *BMC Health Services Research*.

[5] Pfaff H., Hammer A., Ernstmann N., Kowalski C., Ommen O. (2009). Sicherheitskultur: Definition, Modelle Und Gestaltung. Safety Culture: Definition, Models, and Design. *Zeitschrift für Evidenz, Fortbildung und Qualität im Gesundheitswesen*.

[6] Cooper Ph D M. D. (2000). Towards a Model of Safety Culture. *Safety Science*.

[7] Garay S., Haeger M., Kühnlein L., Sulmann D., Suhr R. (2023). Interventions to Enhance Safety Culture for Nursing Professionals in long-term Care: a Systematic Review. *International Journal of Nursing Studies Advances*.

[8] Guldenmund F. W. (2000). The Nature of Safety Culture: a Review of Theory and Research. *Safety Science*.

[9] Garay S., Haeger M., Kühnlein L., Möhr N., Sulmann D. (2022). Sicherheitskultur in Der Ambulanten Pflege. *Safety Culture in Outpatient Care*.

[10] Han Y., Kim J. S., Seo Y. (2020). Cross-Sectional Study on Patient Safety Culture, Patient Safety Competency, and Adverse Events. *Western Journal of Nursing Research*.

[11] Hessels A. J., Paliwal M., Weaver S. H., Siddiqui D., Wurmser T. A. (2019). Impact of Patient Safety Culture on Missed Nursing Care and Adverse Patient Events. *Journal of Nursing Care Quality*.

[12] Li Y., Cen X., Cai X., Temkin-Greener H. (2019). Perceived Patient Safety Culture in Nursing Homes Associated with “Nursing Home Compare” Performance Indicators. *Medical Care*.

[13] Hoffmann B., Hofinger G., Gerlach F. (2009). Wieist Patientensicherheitskultur Messbar? How Is Patient Safety Culture Measurable?. *Zeitschrift für Evidenz, Fortbildung und Qualität im Gesundheitswesen*.

[14] Manser T., Brösterhaus M., Hammer A. (2016). You Can’T Improve what You Don’T Measure: Safety Climate Measures Available in the German-speaking Countries to Support Safety Culture Development in Healthcare. *Zeitschrift fur Evidenz, Fortbildung und Qualitat im Gesundheitswesen*.

[15] Ginsburg L. R., Tregunno D., Norton P. G., Mitchell J. I., Howley H. (2014). ‘Not Another Safety Culture Survey’: Using the Canadian Patient Safety Climate Survey (Can-PSCS) to Measure Provider Perceptions of PSC Across Health Settings. *BMJ Quality and Safety*.

[16] Marshall M., Cruickshank L., Shand J. (2017). Assessing the Safety Culture of Care Homes: A Multimethod Evaluation of the Adaptation, Face Validity and Feasibility of the Manchester Patient Safety Framework. *BMJ Quality and Safety*.

[17] Sorra J., Franklin M., Streagle S. (2008). *Nursing Home Survey on Patient Safety Culture*.

[18] Singer S., Kitch B. T., Rao S. R. (2012). An Exploration of Safety Climate in Nursing Homes. *Journal of Patient Safety*.

[19] Bethune R. M., Ball S., Doran N. (2023). How Safety Culture Surveys Influence the Quality and Safety of Healthcare Organisations. *Cureus*.

[20] Świtalski J., Wnuk K., Tatara T. (2022). Interventions to Increase Patient Safety in Long-Term Care Facilities—Umbrella Review. *International Journal of Environmental Research and Public Health*.

[21] Pacenko C. d L., Figueiredo K. C., Nunes E., Cruchinho P., Lucas P. (2024). Mapping Strategies for Strengthening Safety Culture: A Scoping Review. *Healthcare*.

[22] Dhollande S., Taylor A., Meyer S., Scott M. (2021). Conducting Integrative Reviews: A Guide for Novice Nursing Researchers. *Journal of Research in Nursing*.

[23] Elsbach K. D., van Knippenberg D. (2020). Creating High-Impact Literature Reviews: An Argument for Integrative Reviews. *Journal of Management Studies*.

[24] Hopia H., Latvala E., Liimatainen L. (2016). Reviewing the Methodology of an Integrative Review. *Scandinavian Journal of Caring Sciences*.

[25] Whittemore R., Knafl K. (2005). The Integrative Review: Updated Methodology. *Journal of Advanced Nursing*.

[26] Liberati A., Altman D. G., Tetzlaff J. (2009). The PRISMA Statement for Reporting Systematic Reviews and Meta-Analyses of Studies That Evaluate Healthcare Interventions: Explanation and Elaboration. *BMJ*.

[27] Higgins J. P. T., Savović J., Page M. J., Sterne J. A. (2019). Revised Cochrane Risk-of-Bias Tool for Randomized Trials. *Template for Completion*.

[28] Cochrane Deutschland (2017). Arbeitsgemeinschaft Der Wissenschaftlichen Medizinischen Fachgesellschaften—Institut Für Medizinisches Wissensmanagement. *Bewertung Von Systematischen Übersichtsarbeiten: Ein Manual Für Die Leitlinienerstellung*.

[29] Downes M. J., Brennan M. L., Williams H. C., Dean R. S. (2016). Development of a Critical Appraisal Tool to Assess the Quality of Cross-Sectional Studies (AXIS). *BMJ Open*.

[30] Lockwood C., Munn Z., Porritt K. (2015). Qualitative Research Synthesis: Methodological Guidance for Systematic Reviewers Utilizing Meta-Aggregation. *International Journal of Evidence-Based Healthcare*.

[31] Engle R. L., Gillespie C., Clark V. A., McDannold S. E., Kazi L. E., Hartmann C. W. (2023). Factors Differentiating Nursing Homes With Strong Resident Safety Climate: A Qualitative Study of Leadership and Staff Perspectives. *Journal of Gerontological Nursing*.

[32] He H., Yu P., Li L. (2020). Patient Safety Culture and Obstacles to Adverse Event Reporting in Nursing Homes. *Journal of Nursing Management*.

[33] Kim K. A., Lee J., Kim D., Min D. (2022). Patient Safety Measurement Tools Used in Nursing Homes: A Systematic Literature Review. *BMC Health Services Research*.

[34] Lee Y., Cho E. (2022). Predictors of Patient Safety Activities Among Registered Nurses and Nurse Aides in Long-Term Care Facilities: Cross-Sectional Study. *BMC Geriatrics*.

[35] Liukka M., Hupli M., Turunen H. (2021). Differences Between Professionals’ Views on Patient Safety Culture in Long-Term and Acute Care? A Cross-Sectional Study. *LHS*.

[36] Quach E. D., Kazis L. E., Zhao S. (2021). Organizational Readiness to Change as a Leverage Point for Improving Safety: A National Nursing Home Survey. *BMC Health Services Research*.

[37] Quach E. D., Kazis L. E., Zhao S., McDannold S. E., Clark V. A., Hartmann C. W. (2021). Relationship Between Work Experience and Safety Climate in Veterans Affairs Nursing Homes Nationwide. *Journal of Patient Safety*.

[38] Quach E. D., Kazis L. E., Zhao S. (2021). Safety Climate Associated With Adverse Events in Nursing Homes: A National VA Study. *Journal of the American Medical Directors Association*.

[39] Rand S., Smith N., Jones K., Dargan A., Hogan H. (2021). Measuring Safety in Older Adult Care Homes: A Scoping Review of the International Literature. *BMJ Open*.

[40] Scott J., Sykes K., Waring J. (2024). Systematic Review of Types of Safety Incidents and the Processes and Systems Used for Safety Incident Reporting in Care Homes. *Journal of Advanced Nursing*.

[41] Seljemo C., Viksveen P., Ree E. (2020). The Role of Transformational Leadership, Job Demands and Job Resources for Patient Safety Culture in Norwegian Nursing Homes: A Cross-Sectional Study. *BMC Health Services Research*.

[42] Serre N., Espin S., Indar A., Bookey-Bassett S., LeGrow K. (2022). Long-Term Care Nurses’ Experiences With Patient Safety Incident Management: A Qualitative Study. *Journal of Nursing Care Quality*.

[43] Teigné D., Mabileau G., Moret L., Terrien N. (2021). What Is the Level of Safety Culture in French Nursing Homes? The EHPAGE Study. *BMC Health Services Research*.

[44] Teigné D., Mabileau G., Lucas M., Moret L., Terrien N., Alipour J. (2022). Safety Culture in French Nursing Homes: A Randomised Controlled Study to Evaluate the Effectiveness of a Risk Management Intervention Associated With Care. *PLoS One*.

[45] Temkin-Greener H., Cen X., Li Y., Meeks S. (2020). Nursing Home Staff Turnover and Perceived Patient Safety Culture: Results From a National Survey. *The Gerontologist*.

[46] Wang Z., Shi Y., Shao L., Xie X., Li X., Zhang J. (2024). Adverse Event Reporting Attitude and Its Individual and Organizational Predictors Among Nursing Staff: A Multisite Study in Chinese Nursing Homes. *Geriatric Nursing*.

[47] Yamamoto E., Hatanaka K., Tanaka T. (2023). Collaborative Approach to Safety in Nursing Homes: Perspectives of Caregivers and Nurses. *ACCS Official Conference Proceedings*.

[48] Gartshore E., Waring J., Timmons S. (2017). Patient Safety Culture in Care Homes for Older People: A Scoping Review. *BMC Health Services Research*.

[49] European Network for Patient Safety-EunetPaS (2010). Patient Safety Culture Instruments Used in Member States. *European Society for Quality in Healthcare ‐ Office for Quality Indicators*.

[50] Sorra J., Nieva V. (2004). Hospital Survey on Patient Safety Culture. *Agency for Healthcare Research and Quality*.

[51] Viksveen P., Røhne M., Grut L., Cappelen K., Wiig S., Ree E. (2022). Psychometric Properties of the Full and Short Version Nursing Home Survey on Patient Safety Culture (NHSOPSC) Instrument: A Cross-Sectional Study Assessing Patient Safety Culture in Norwegian Homecare Services. *BMJ Open*.

[52] Schein E. H. (2010). *Organizational Culture and Leadership*.

